# Artificial intelligence in the lab: ask not what your computer can do for you

**DOI:** 10.1111/1751-7915.13317

**Published:** 2018-09-24

**Authors:** Dick de Ridder

**Affiliations:** ^1^ Bioinformatics Group Wageningen University & Research Droevendaalsesteeg 1 6708 PB Wageningen The Netherlands

In 1957, Herbert Simon, a pioneer of artificial intelligence, predicted that a computer would be the world chess champion within 10 years. It took somewhat longer, but he was eventually proven right when IBM's Deep Blue computer beat Gary Kasparov in 1997. This major breakthrough in artificial intelligence was, in a way, also one of the last successes of what was known as ‘good old‐fashioned AI’: the idea that to mimic and understand human intelligence, computers should represent knowledge as symbols and apply reasoning and rules to infer new knowledge. This notion had been criticized for some time already (Dreyfus and Dreyfus, 1992) and over the years gradually lost ground to another approach, machine learning, in which statistical models were fitted to data to derive patterns and correlations. Widely known systems that fit this category include Watson, which successfully competed against the best human players in the Jeopardy! general knowledge quiz, and Google's AlphaGo, which in 2017 beat the reigning world champion at the game of Go. In other settings as well, machine learning progressed. In 2012, it was demonstrated how an extremely large neural network, AlexNet, could be trained to recognize images in 1000 different categories, an atpproach that became known as deep learning (LeCun *et al*., 2015). Machine learning and deep learning are now routinely used by companies such as Google, Facebook, Amazon and Tesla in products ranging from automated translation and home automation to self‐driving cars.

In biology, machine learning has likewise found its use. Large volumes of ‐omics data can now routinely be measured and are used to infer biological function. Many bioinformatics algorithms under the hood rely on statistical models trained on such data to predict – often from nucleotide or amino acid sequences – the structure of genes, the function, location, domain content and secondary structure of proteins, the interactions of proteins with other proteins and DNA, phenotypes, etc. Deep learning has been applied to biological data as well, predicting, among others, protein–DNA interactions (DeepBind), gene regulation (DeepChrome) and variant effects (DeepSEA; Min *et al*., 2017).

A particularly interesting application of machine learning is one where the computer is not only able to predict the function of a sequence or set of sequences, but to (re)design sequences to achieve a certain desired function. This will make it possible to design bespoke regulatory elements, molecules and interactions on‐demand, the building blocks needed to fulfil the promise of synthetic biology to engineer microbial machines (Vickers, 2017). Algorithms have been developed to directly predict a sequence given a function, for example inferring an amino acid sequence most likely to fold into a desired three‐dimensional structure (O'Connell *et al*., 2018) or a DNA sequence most likely to bind a certain protein (Killoran *et al*., 2017). Alternatively, search algorithms can iteratively try mutating a given sequence and keep those changes considered beneficial by a function predictor (Guimaraes *et al*., 2014), for example to improve protein production (van den Berg *et al*., 2014).

In essence, such sequence (re)design approaches are similar to the AlphaGo setup, in which deep learning networks are used to evaluate Go board positions and moves, based on which a search algorithm decides the next best move to make. In both cases, the search space (the number of mutations or moves to consider) is extremely high‐dimensional: in the order of 20^400^ for a 400‐amino acid protein design, and 250^150^ for a game Go. Deep learning can still successfully learn to predict the value of previously unseen input in such huge spaces, but it requires two things: massive computational resources and extremely large sets of examples. Both are now available for many applications; deep learning was in large part made possible by the advent of affordable GPU‐based devices and is most successful in areas where large data sets have become available. For example, AlexNet was trained on over a million images labelled by crowdsourcing through the Internet, and Google Translate is based on millions of online documents.

In biology, we are not quite there yet. Even though many measurement sets are generated, for a single specific problem very large data sets are not often available; in particular, reliable outputs are often lacking. For example, we know the sequences of millions of proteins, but only have experimentally verified functions of a few hundreds of thousands, and often only in model organisms. However, the tide is turning, combining the possibilities offered by cheap DNA synthesis and sequencing in protocols to measure sequence–function relations at unprecedented scales. In so‐called deep mutational scanning, massively parallel reporter assays (MPRAs) or multiplexed assays for variant effects (MAVEs), thousands to hundreds of thousands of sequence variants are generated and their effect on transcription, translation or function is assessed (Gasperini *et al*., 2016). If such data sets are mainly measured to test specific hypotheses on the effects of limited levels of variation, they may not be useful to train machine learning models that can generalize, but this is changing as well. In recent examples, researchers fit models to 244 000 variants of a gene in *Escherichia coli* to learn about the influence of sequence composition on translation (Cambray *et al*., 2017) and trained a deep learning network to predict protein expression in *Saccharomyces cerevisiae* from a set of 500 000 random 50‐nt 5′ UTR sequences (Cuperus *et al*., 2017).

So it seems safe to predict that, like in many other areas, high‐throughput modelling – machine learning on massive data sets specifically generated to train models – will become standard practice in the near future. An important question then is what to research, i.e. what sequence variants to investigate. The full search space for cellular genomes is immense, even for minimal genomes – there are 4^580,000^ possible genomes of the size of *Mycoplasma genitalium*. Of course, evolution has already explored part of this space, and extant genomes provide a good starting point. To proceed, we can actually learn from the AlphaGo approach. Initially, this system was trained on a large database of human Go games, after which it improved quickly by playing games against versions of itself (Silver *et al*., 2016) (later versions even started from scratch). This form of ‘on‐the‐job training’ is called reinforcement learning and is applicable in situations such as games, where a series of actions is eventually rewarded (if it leads to a win) or penalized (if it causes a loss).

In biological experiments, we generally cannot as easily declare victory, but we can use the systems biology approach of cycling between experimentation and modelling to see which sequences, when tested, are most likely to improve the model. In artificial intelligence, this is called active learning, and it has some similarity to the way in which we as humans learn as infants: we get some help from parents and teachers, but mainly model the world around us by exploring it and interacting with it. Ideally then, we would recreate such an environment for our machine learning algorithms in the laboratory, where we start with an initial ‘infant’ model of a certain regulatory system or protein function and let the computer decide what sequence designs to try out – a deep learning version of the ‘robot scientist’ (King *et al*., 2009). Microbes are ideal organisms for such an approach, given the ease and speed with which they can be grown and genetically manipulated. Combined with laboratory automation, many microbial experiments can (soon) be performed with minimal human intervention, ranging from strain construction and screening, such as operated by Amyris, Gingko, Transcriptic, etc., to full‐genome engineering or even the design of microbial ecologies. As demonstrated by Zymergen, in some cases it is already feasible to simply define our engineering goals and let the robots figure out how to achieve it (Bohannon, 2017). In such a setting, would we become mere servants to our new robotic overlords? I do not believe so – if anything, the increased speed and scope of experimentation will free up our creativity and allow us take microbial machinery to places we could not imagine going today (Timmis *et al*., 2017).

One important question remains, though: while this makes for great engineering, where does it leave science? Interestingly, a similar discussion is going on in artificial intelligence. At a 2011 meeting at MIT, Noam Chomsky – a veteran computational linguist, among others – dismissed machine learning‐based AI as follows: ‘There is a notion of success which has developed in computational cognitive science in recent years which I think is novel in the history of science. It interprets success as approximating unanalysed data’. In other words, although we may be able to predict (or engineer) some phenomenon, it does not mean that we actually understand it – especially if we use a ‘black‐box’ model deriving correlations from data, such as a deep learning network. I would counter that while this is true, the same can be said of any model in science (paraphrasing George Box). In systems biology, models are not the end goal, but tools to capture our current knowledge, combine it with new data and derive creative new hypotheses on causes and effects to be verified experimentally. And while a machine learning model may be harder to interpret than a differential equation, it is often quite possible and can be highly informative (Breiman, 2001).

In summary, then, like in any other endeavour, artificial intelligence will likely have a major impact on the way we work as biologists and biotechnologists, by closing the loop between experimentation and analysis. Increasingly we will measure data specifically to feed machine learning models and let such models guide our experimentation by proposing new sequences to synthesize, new perturbations to apply, new conditions to try, etc. As a consequence, more than ever advances in engineering and science will become mutually interdependent. And luckily, there is still room for human engineers to create these tools, and for human scientists to make sense of it all.

## Conflict of interest

None declared.

## References

[mbt213317-bib-0001] Bohannon, J. (2017) The cyberscientist. Science 357: 18–21. 28684480 10.1126/science.357.6346.18

[mbt213317-bib-0002] Breiman, L. (2001) Statistical modeling: the two cultures. Stat Sci 16: 199–231.

[mbt213317-bib-0003] Cambray, G. , Guimaraes, J.C. and Arkin, A.P. (2017) Massive factorial design untangles coding sequences determinants of translation efficacy. bioRxiv, 10.1101/208801

[mbt213317-bib-0004] Cuperus, J.T. , Groves, B. , Kuchina, A. , Rosenberg, A.B. , Jojic, N. , Fields, S. , and Seelig, G. (2017) Deep learning of the regulatory grammar of yeast 5′ untranslated regions from 500,000 random sequences. Genome Res 27: 2015–2024. 29097404 10.1101/gr.224964.117 PMC5741052

[mbt213317-bib-0005] Dreyfus, H.L. , and Dreyfus, S.E. (1992) What artificial experts can and cannot do. AI and Soc 6: 18–26.

[mbt213317-bib-0006] Gasperini, M. , Starita, L. , and Shendure, J. (2016) The power of multiplexed functional analysis of genetic variants. Nat Protoc 11: 1782–1787. 27583640 10.1038/nprot.2016.135 PMC6690347

[mbt213317-bib-0007] Guimaraes, J.C. , Rocha, M. , Arkin, A.P. , and Cambray, G. (2014) D‐Tailor: automated analysis and design of DNA sequences. Bioinformatics 30: 1087–1094. 24398007 10.1093/bioinformatics/btt742 PMC3982154

[mbt213317-bib-0009] Killoran, N. , Lee, L.J. , Delong, A. , Duvenaud, D.K. and Frey, B.J. (2017) Generating and designing DNA with deep generative models. arXiv 1712.06148v1.

[mbt213317-bib-0010] King, R.D. , Rowland, J. , Oliver, S.G. , Young, M. , Aubrey, W. , Byrne, E. , *et al* (2009) The automation of science. Science 324: 85–89. 19342587 10.1126/science.1165620

[mbt213317-bib-0011] LeCun, Y. , Bengio, Y. , and Hinton, G. (2015) Deep learning. Nature 521: 436–444. 26017442 10.1038/nature14539

[mbt213317-bib-0012] Min, S. , Lee, B. , and Yoon, S. (2017) Deep learning in bioinformatics. Brief Bioinform 18: 851–869. 27473064 10.1093/bib/bbw068

[mbt213317-bib-0013] O'Connell, J. , Li, Z. , Hanson, J. , Heffernan, R. , Lyons, J. , Paliwal, K. , *et al* (2018) SPIN2: predicting sequence profiles from protein structures using deep neural networks. Proteins 86: 629–633. 29508448 10.1002/prot.25489

[mbt213317-bib-0014] Silver, D. , Huang, A. , Maddison, C.J. , Guez, A. , Sifre, L. , van den Driessche, G. , *et al* (2016) Mastering the game of Go with deep neural networks and tree search. Nature 529: 484–489. 26819042 10.1038/nature16961

[mbt213317-bib-0015] Timmis, K. , de Lorenzo, V. , Verstraete, W. , Ramos, J.L. , Danchin, A. , Brüssow, H. , *et al* (2017) The contribution of microbial biotechnology to economic growth and employment creation. Microb Biotechnol 10: 1137–1144. 28868756 10.1111/1751-7915.12845 PMC5609265

[mbt213317-bib-0016] van den Berg, B.A. , Reinders, M.J.T. , van der Laan, J.M. , Roubos, J.A. , and de Ridder, D. (2014) Protein redesign by learning from data. Protein Eng Des Sel 27: 281–288. 25082898 10.1093/protein/gzu031

[mbt213317-bib-0017] Vickers, C. (2017) Bespoke design of whole‐cell microbial machines. Microb Biotechnol 10: 35–36. 27860240 10.1111/1751-7915.12460 PMC5270718

